# Impact of film thickness in laser-induced periodic structures on amorphous Si films

**DOI:** 10.1007/s12200-023-00071-6

**Published:** 2023-06-20

**Authors:** Liye Xu, Jiao Geng, Liping Shi, Weicheng Cui, Min Qiu

**Affiliations:** 1 College of Optical Science and Engineering, Zhejiang University, Hangzhou, 310058 China; 2 Key Laboratory of 3D Micro/Nano Fabrication and Characterization of Zhejiang Province, School of Engineering, Westlake University, Hangzhou, 310024 China; 3 Key Laboratory of Coastal Environment and Resources of Zhejiang Province (KLaCER), School of Engineering, Westlake University, Hangzhou, 310024 China; 4 Institute of Advanced Technology, Westlake Institute for Advanced Study, Hangzhou, 310024 China; 5 Wuhan National Laboratory for Optoelectronics, Huazhong University of Science and Technology, Wuhan, 430074 China

**Keywords:** Laser-induced periodic surface structures (LIPSS), Ultrafast optoelectronics, Laser nanofabrication, Quasi-cylindrical waves

## Abstract

We report self-organized periodic nanostructures on amorphous silicon thin films by femtosecond laser-induced oxidation. The dependence of structural periodicity on the thickness of silicon films and the substrate materials is investigated. The results reveal that when silicon film is 200 nm, the period of self-organized nanostructures is close to the laser wavelength and is insensitive to the substrates. In contrast, when the silicon film is 50 nm, the period of nanostructures is much shorter than the laser wavelength, and is dependent on the substrates. Furthermore, we demonstrate that, for the thick silicon films, quasi-cylindrical waves dominate the formation of periodic nanostructures, while for the thin silicon films, the formation originates from slab waveguide modes. Finite-difference time-domain method-based numerical simulations support the experimental discoveries.

**Graphical abstract:**

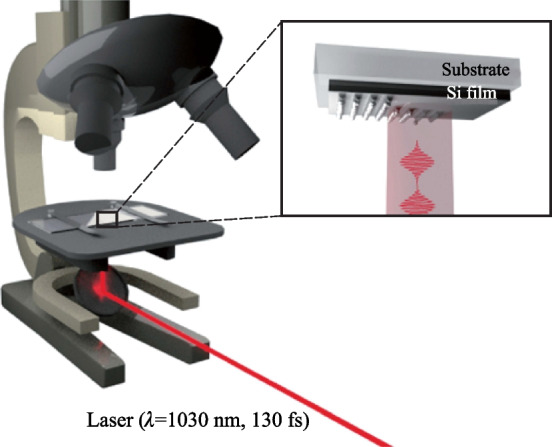

**Supplementary Information:**

The online version contains supplementary material available at 10.1007/s12200-023-00071-6.

## Introduction

Due to its accessibility and low cost, silicon (Si) has been widely adopted in microelectronics and optoelectronics. Si exhibits unique advantages in photonic applications, from single nanoresonators to functional photonic metasurfaces and waveguides [1–6]. The widespread applications of Si in nanophotonics were initially triggered by the observation of electric and magnetic multi-polar Mie-type resonances in all-dielectric nanoparticles with high refractive index. Among the common dielectric materials, Si has emerged as a popular candidate. This is not only because of its high refractive index and low intrinsic loss in the telecommunication spectral range, but also due to its paramount technological relevance with nanofabrication.

Conventional nanofabrication techniques are generally a combination of electron-beam lithography and photolithography. The former is used to write nanoscale photomasks, and the latter is used to replicate those masks. However, in recent years, to pursue low-cost, large-scale and convenient nanofabrication, processing materials by femtosecond laser pulses has attracted considerable interest. For instance, the inscription of Si functional structures, including gratings [7, 8], waveguides [9] and microfluidic channels [10], has been implemented by direct laser writing.

However, direct laser writing is facing the problem of low fabrication speed As an alternative, laser-induced periodic surface structures (LIPSS) [11–14] exhibit much faster manufacturing speed up to the order of m^2^/s [12]. LIPSS was first observed by Birnbaum in 1965 [15], and attracted wide attention with the development of femtosecond lasers since the 1990s. From the standpoint of practical applications, LIPSS not only offers a low-cost, robust, single-step and flexible approach for the large-scale fabrication of periodic nanostructures but also can overcome the optical diffraction limit. More importantly, LIPSS can be applied on the surface of almost any material, including semiconductors [16, 17], dielectrics [18], and metals [19, 20]. Therefore, it has been considered an appealing technique and holds the potential to overcome the barrier between macro and nano-manufacturing.

Although LIPSS has been investigated for over half a century, many fundamental questions still exist. An ongoing research line is achieving large-scale processing with long-range uniformity [21]. The long-range uniformity can be influenced by several factors, including the decay propagation distance of surface electromagnetic waves [22], the electron–phonon coupling coefficient of materials [23], the scanning speed and direction of laser beams [24, 25], surface chemical compositions [26], residual heat and redeposited surface debris [27], etc. In recent years, different methods have been demonstrated to improve the long-range uniformity of LIPSS, such as ablation cooling [27], intense ablation [28], laser beam shaping [29], electron dynamic control by dual pulses [30], and laser-induced oxidation on thin films [31–33].

Among these approaches, the oxidative LIPSS is a relatively new type. Compared with ablative LIPSS, the oxidative approach has a much lower laser fluence threshold and thus results in less residual heat and less surface debris. As a result, oxidative LIPSS generally exhibits more ordered structures, i.e., there are no phase shifts and deviations in the spatial period [31, 34]. Recently, in terms of femtosecond laser-induced oxidation, large-scale periodic nanostructures on Si thin films with high uniformity and high fabrication speed have been demonstrated by several groups [35, 36].

In general, ultrafast laser-induced low-spatial frequency LIPSS (LSFL) with Λ > *λ*/2, where Λ and *λ* denote the period of LIPSS and laser wavelength respectively, can be divided into two categories according to the orientation of the periodic structure with respect to the polarization direction of the laser light and the period with respect to laser wavelength. For strongly absorbing materials such as metals and Si, LIPSS exhibits spatial periods close to laser wavelength and orientation perpendicular to the polarization direction of the laser, which is classified as LSFL-I. The periodic structures are typically induced by surface plasmon polaritons (SPPs) [12]. For transparent materials such as wide-bandgap dielectrics, LIPSS exhibits a sub-wavelength period Λ ∼ *λ*/*n*, with* n* being the material’s refractive index, and orientation is parallel to the polarization direction of the laser beam. This case is classified as LSFL-II, which is related to the so-called radiation remnants.

However, classical oxidative LIPSS on thin films differs from the classifications mentioned above. The periodic structure aligns parallel to polarization direction of the laser light, and its period depends on the thickness of thin films. Dostovalov et al. [35] found that when the thickness of amorphous Si (a-Si) film exceeds 100 nm, the spatial period is close to the laser wavelength, whereas when the thickness decreases to < 100 nm, the period abruptly reduces to < 600 nm. This abrupt variation was qualitatively attributed to the fact that the effective refractive index of Si is related to its thickness. Nevertheless, a deep insight into the influence of a-Si thickness on periodicity still needs to be disclosed.

## Experimental setups

**Fig. 1:**
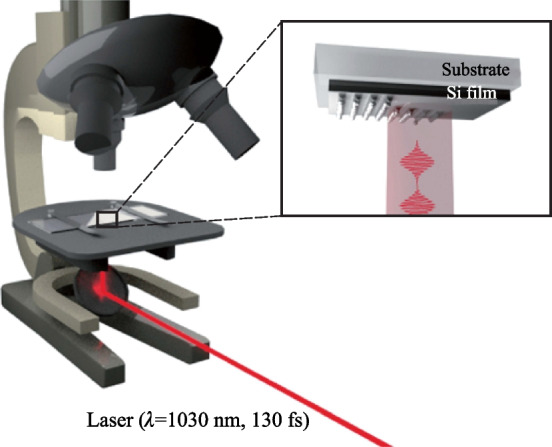
Schematic illustration of the experimental setup for laser processing and in situ microscopic observation

In this article, we experimentally and numerically investigated the dependence of laser-induced structural periodicity on the thickness of a-Si films from the perspective of surface electromagnetic waves. We used a magnetron sputtering system (ULVAC CS200Z) operating at room temperature to deposit 50 and 200 nm thick a-Si films on sapphire (Al_2_O_3_), fused silica (SiO_2_) and crystalline Si (c-Si) substrates. The thickness of the a-Si films was measured by a profiler (Stylus). As shown in Fig. 1, a femtosecond laser (Amplitude Tangerine), with a central wavelength of 1030 nm, pulse duration of 130 fs, and a repetition rate of 5 kHz was loosely focused onto the samples by a lens (focal length = 20 cm). The spot diameter was measured to be 80 µm by a beam profiler. The laser beam irradiated the sample from the a-Si film side and the growth of nanostructures was observed by a microscope from the substrate side when the substrate is transparent. The laser fluence at the sample surface was evaluated to be 20 mJ/cm^2^. For the 50 nm thick a-Si film, the scan velocity was 10 µm/s; for the 200 nm thick a-Si film, the scan velocity was 2 µm/s.

## Results

**Fig. 2:**
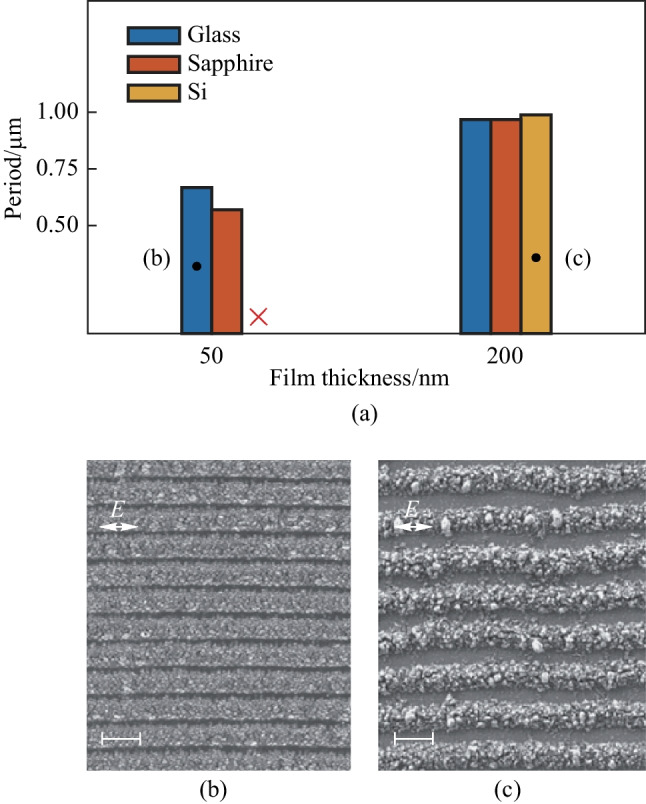
**a** Dependence of LIPSS period on film thicknesses and substrates. SEM images of stationary irradiation-induced periodic nanostructures on different thicknesses of a-Si film: **b** 50 nm on glass and **c** 200 nm on c-Si. Scale bars: 1 µm

Figure 2a lists the LIPSS period for different a-Si film thickness and substrates. Figure 2b and c show the representative scanning electron microscopy (SEM) images of the LIPSS on a 50 nm thick a-Si film on glass and a 200 nm thick a-Si film on c-Si. We found that the orientation of periodic structures was independent of the a-Si thickness, and parallel to the polarization direction of the incident laser, which was in agreement with the feature of typical oxidative LIPSS [31]. However, their periods were evidently related to the Si thickness. When the thickness of the a-Si film was 50 nm on the glass substrate, the period could be measured from the high-resolution SEM images as 578 nm. Furthermore, we found that for the case of 50-nm-thick a-Si, the LIPSS period was dependent on the materials of the substrate. Especially, when the substrate was c-Si, we could not obtain regular periodic structures. When the a-Si film thickness was 200 nm, the period increased up to 990 nm, and in this scenario, the period was independent of the substrate materials.

As shown in Fig. S1, to understand the dependence of LIPSS period on a-Si thickness, as well as on the substrate materials, we carried out numerical simulations based on FDTD for different substrates and different a-Si film thicknesses to investigate the distribution of electromagnetic waves.

**Fig. 3:**
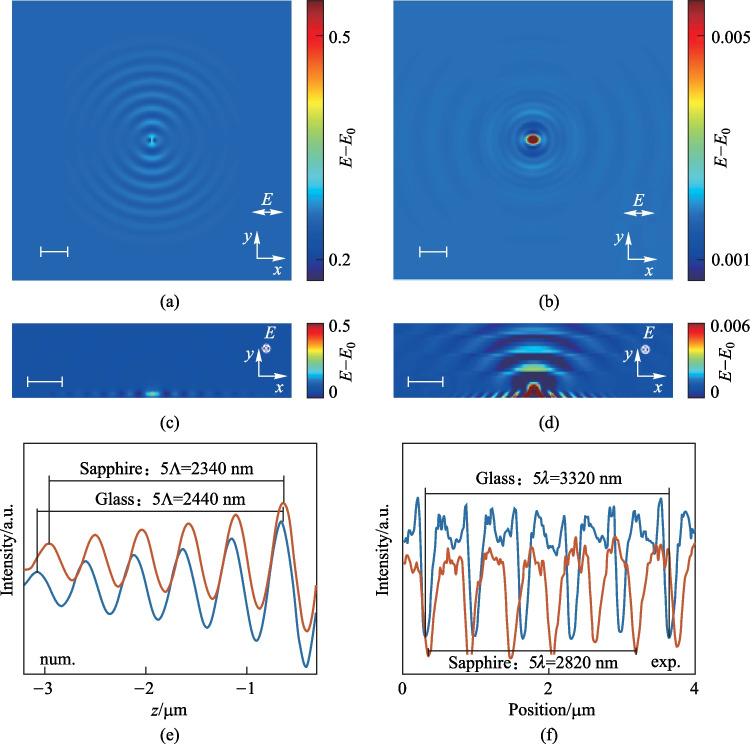
FDTD-based simulation results of 50 nm a-Si on the glass substrate (**a**), and the c-Si substrate (**b**), monitored in the *x*–*y* plane of interface between Si film and air, and in the corresponding *y*–*z* plane (**c**, **d**). The distribution of electric field strength at *x* = 0 of FDTD simulation results on glass and sapphire substrate (**e**). and the gray intensity distribution of SEM images of LIPSS of 50 nm a-Si film on silicon and sapphire substrates (**f**). Scale bars: 1 µm

Figure 3 shows simulation results of 50-nm-thick a-Si films on various substrates. A sphere-shaped silicon oxide particle with a diameter of 100 nm was half immersed into the Si film, which acted as a scatter. The light polarized along the *x*-axis. Figure 3a and c depict the electric field distribution at the air-Si interface and the cross-sectional *y–z* plane, respectively, when the substrate was glass. The scattering waves, interfering with the incident light, gave rise to interference fringes that align along the polarization direction of light (Fig. 3a). Furthermore, the scattered waves were bounded within the Si film, as shown in Fig. 3c, indicating that the slab waveguide modes (WGMs) were excited in the Si film. The wavelength of the WGMs was sensitive to the refractive index of substrate material, as shown in Fig. 3e. Sapphire, which exhibited a higher refractive index, led to a smaller wavelength of WGMs compared to glass, and this qualitatively agreed with the experimental results as plotted in Fig. 3f. We found that the measured periods were slightly greater than the simulated ones, because the transient refractive index of Si was reduced due to nonlinear excitation of carriers under femtosecond laser irradiation. The decrease of the refractive index then resulted in the increase of WGMs’ wavelength. It was challenging to precisely predict the LIPSS period from the numerical simulations that only consider the linear response. When the substrate was replaced by a c-Si wafer whose refractive index approaches a-Si, the interference pattern nearly disappeared (Fig. 3b and d). This occurred because, in this case, substantial leakage of the light field existed at the interface of c-Si and a-Si and WGMs were too weak to support the generation of oxidized LIPSS. In addition, as we discuss in the supplementary materials, the oxidized particles scattered the incident light field and the size of these particles significantly affected the intensity of the excited quasi-cylindrical waves (QCWs). When the film was relatively thin (50 nm), the size of the oxidized particles that could be grown was limited. As a result, neither WGMs nor QCWs could successfully produce a large area of ordered LIPSS.

**Fig. 4:**
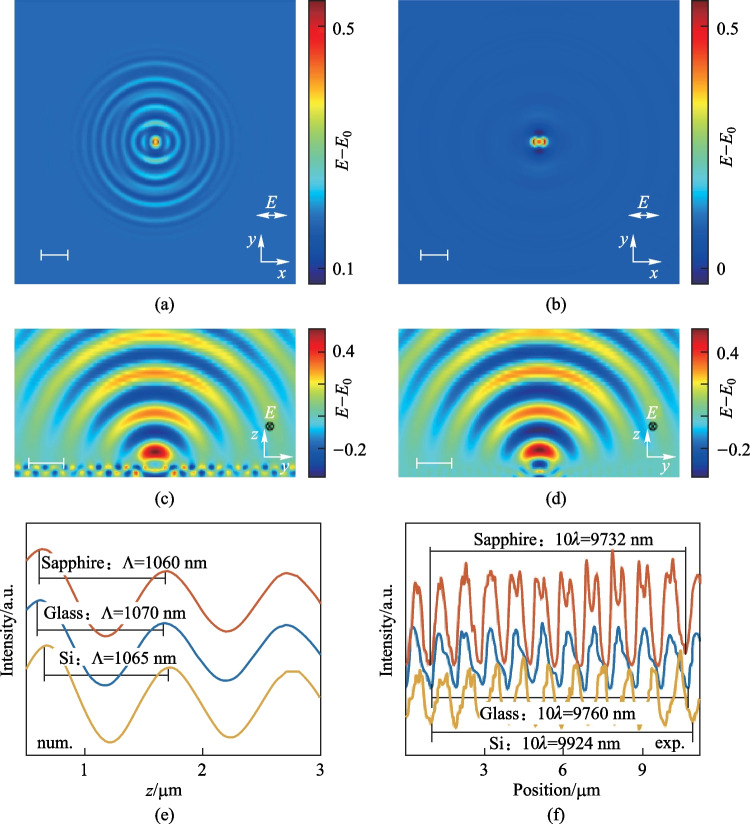
FDTD-based simulation results of 200 nm a-Si on a sapphire substrate (**a**) and on a c-Si substrate (**b**) monitored in the *x–y* plane of interface between Si film and air, and in corresponding *y–z* plane when the particle grows into a ridge (**c**, **d**). The distribution of electric field strength at *x* = 0 of FDTD simulation results (**e**) and the gray intensity distribution of SEM images of LIPSS of 200 nm a-Si film on silicon, sapphire and Si substrates (**f**). Scale bars: 1 µm

In the next step, we performed numerical simulations of 200-nm-thick a-Si films. To closely match the experimental situation, a larger-sized scattering source was used when the film thickness became thicker. A sphere-shaped SiO_2_ particle with a diameter of 400 nm was half embedded into the a-Si film. As shown in Fig. 4a, the interfacial electromagnetic waves were more complicated compared to those for the case of 50-nm-Si films depositing on glass (Fig. 3a). In addition to the standing wave that was along the light polarization direction with a period of 600 nm, we also observed another standing wave that had a period of ~ *λ*_laser_. When the substrate was replaced by c-Si, only the diffraction field with a period of ~ *λ*_laser_ was observed, as shown in Fig. 4b. Therefore, it was certain that the other EM wave mode dominated and induced the formation of LIPSS when the film thickness was 200 nm and the period of the EM field distribution was close to the period of the incident wave. The process in which this electromagnetic mode (QCWs) induced the growth of oxidative LIPSS has been elucidated by Geng et al. [37]. In order to observe the distribution of quasi-cylindrical waves more clearly, we also investigated the cross-sectional field distribution when the scattering source was an oxidized ridge. As shown in Fig. 4c and d, it could be observed that the quasi-cylindrical waves propagated along the Si-air surface and also radiated into the air [37, 38]. The quasi-cylindrical waves had a wavelength close to *λ*_laser_ and was independent of the substrate materials (Fig. 4e). In experiment, we confirmed that the period of LIPSS on 200 nm thick a-Si were nearly independent of the substrate materials (Fig. 4f). The quasi-cylindrical waves propagated in directions both parallel and perpendicular to the polarization direction of the laser. Therefore, in the case that the formation of periodic structure was dominated by quasi-cylindrical waves, the orientation of the periodic structure was determined by near-field scattering in the vicinity of the nanoparticles [37]. Due to the near field, the oxided particles would gradually grow into ridges, and the first intensity peak of the quasi-cylindrical wave made more ridges gradually grow in the neighborhood. According to the equation derived by Geng et al. [37], the distance between the position of the first intensity peak and the scattering source was close to the wavelength of the incident light, and our experiment and simulation results were consistent with this deduction, as shown in Fig. 4e and f.

## Conclusions

In summary, we have successfully fabricated LIPSS using a femtosecond pulsed laser, and two different spatial periods of the periodic structures was obtained on silicon films of different thicknesses. The mechanisms behind the formation of these periodic structure was analyzed, and was found to be due to different electromagnetic modes. According to simulation and experiment results, for LIPSS with different periods produced on 50 nm Si film on sapphire and glass, we believe that the substrate’s refractive index has an effect on the period. The difference in LIPSS periods indicated that the slab waveguide mode dominated the formation of LIPSS in this case. In addition, LIPSS with a period close to the incident wavelength was successfully produced on a 200 nm a-Si film and the dominant mechanism was attributed to the quasi-cylindrical wave. FDTD simulations were also carried out, and consistent results were obtained. By controlling the refractive index of the substrate and the thickness of the a-Si film, the period of the LIPSS could be controlled.

## Supplementary Information

Below is the link to the electronic supplementary material.

Supplementary file 1 (PDF 272 KB)

## Data Availability

The data that support the findings of this study are available from the corresponding author, upon reasonable request.
