# Supplementary material for: Impact of film thickness in laser-induced periodic structures on amorphous Si films
Source: Front Optoelectron. 2023 Jun 20;16(1):16. doi: 10.1007/s12200-023-00071-6 (PMC10281936; doi:10.1007/s12200-023-00071-6)
Supplement: Supplementary file 1 — (PDF 272 KB) [file 12200_2023_71_MOESM1_ESM.pdf]

Supplementary materials

Impact of film thickness in laser-induced periodic structures on amorphous Si films

Liye Xu[1,2,3,4], Jiao Geng[2,4*], Liping Shi[2,4,5], Weicheng Cui[3,4*], Min Qiu[2,4*]

[1]College of Optical Science and Engineering, Zhejiang University, Hangzhou 310058, China.

[2]Key Laboratory of 3D Micro/Nano Fabrication and Characterization of Zhejiang Province, School of Engineering, Westlake University, Hangzhou 310024, China.

[3]Key Laboratory of Coastal Environment and Resources of Zhejiang Province (KLaCER), School of Engineering, Westlake University, Hangzhou 310024, China.

[4]Institute of Advanced Technology, Westlake Institute for Advanced Study, Hangzhou 310024, China.

[5]Wuhan National Laboratory for Optoelectronics, Huazhong University of Science and Technology, Wuhan 430079, China.

*Corresponding author(s). E-mail(s): gengjiao@westlake.edu.cn; cuiweicheng@westlake.edu.cn; qiumin@westlake.edu.cn;

Numerical simulation based on FDTD setup

We used the FDTD-based method to calculate the electric field distribution in the whole space after the interaction between the incident light and the nanoparticles of different a-Si thicknesses and different substrate materials. The refractive index of the a-Si in the experiments was measured by an ellipsometer and was used during the simulation, while the other 3 materials (Al_2O_3, SiO_2, Si)' refractive indices as substrate were from Palik's measurement [1]. There were 3 layers to form the simulation structure, the bottom layer was substrate (sapphire, glass, c-Si) with the thickness of 1180 nm while the middle layer was an a-Si film with a thickness of 50 nm and 200 nm respectively. We used a SiO_2 sphere as the top layer because in the actual experimental process, the electric field is initially distributed due to the oxidized particles, and under the action of the electric field, the SiO2 gradually builds up and grows into regular stripes. In addition, the oxidation process occurs simultaneously at the interface along both the air and amorphous silicon directions, in order to simulate the actual electric field distribution, we let the sphere embed in the a-Si film. To observe the clear distribution of the electric field, we chose silica spheres with a diameter of 100 nm for the 50 nm a-Si film and 400 nm for the 200 nm film thickness. When quasi-cylindrical waves dominated in the 200 nm a-Si film case, the SiO_2 particle grew to a ridge

because of the near-field influence. In order to be consistent with the real situation, we also simulate the case when the SiO_2 sphere was replaced by a cylinder of 400 nm bottom diameter and 6000 nm length embedded in the a-Si layer. As illustrated in Fig. 4(c) and Fig. 4(d), the intensity of the quasi-cylindrical wave is stronger than that of the sphere, and the first peak starts to induce the formation of the next ridge. The simulation area is set as a rectangle of 34 μm * 34 μm * 5 μm. The incident source is a monochromatic plane wave with a wavelength of 1030 nm and is polarized along the x-axis. In order to observe the quasi-cylindrical wave as well as the slab waveguide, two frequency-domain field and power monitors are set to investigate the electric field distribution at the silicon and air interface and at the y-z-plane of the oxidized sphere or cylinder. The SiO_2 sphere or cylinder is placed in the center of the entire simulation area. The simulation time is 1000 fs and we assume that the simulation converges when the auto-off level is below 1.0×10^{-5} and the simulation process does not exceed 90%.

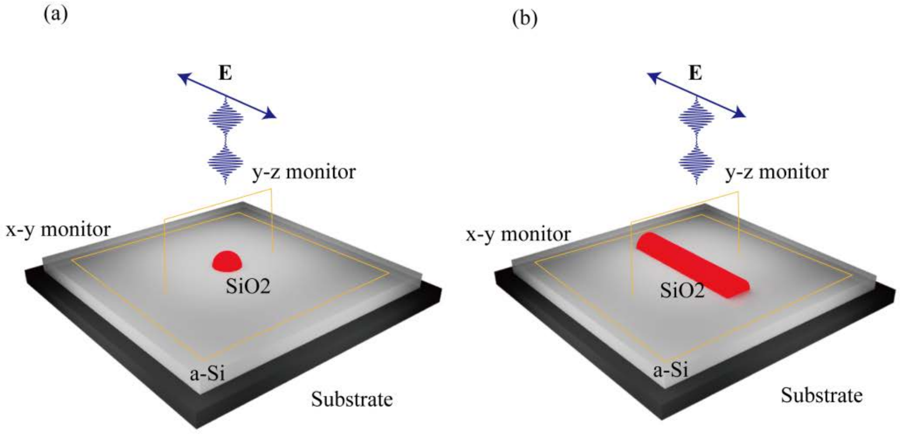

Figure 1 Schematic diagram of FDTD setup when the scatter source is the **(a)** spheric nanoparticle and the **(b)** cylindrical nanowire.

The influence of particle size

During the growth process of oxidative LIPSS, the initial oxide particles on the surface act as a scattering source, allowing an orderly distribution of electromagnetic waves. Under continuous laser irradiation, the particle size increases because of continuous oxidation, but the size is limited by the a-Si film thickness eventually. Regardless of the type of material used for the substrate, it can be observed from the experimental results that when the film thickness of a-Si increases from 50 nm to 200 nm, the quasi-cylindrical waves dominate and the space period of LIPSS is independent of the substrate material. The increase in film thickness implies an increase in oxidation depth so that the size of the particles formed on the surface can grow larger, which leads to an increase in the intensity of the induced quasi-cylindrical waves. As shown in the figure, the numerical simulations show that when the size of the oxide particle as a scattering source increases, the excited quasi-cylindrical waves become stronger. The ordered distribution of quasi-cylindrical waves eventually induces the production of large regular oxidation LIPSS.

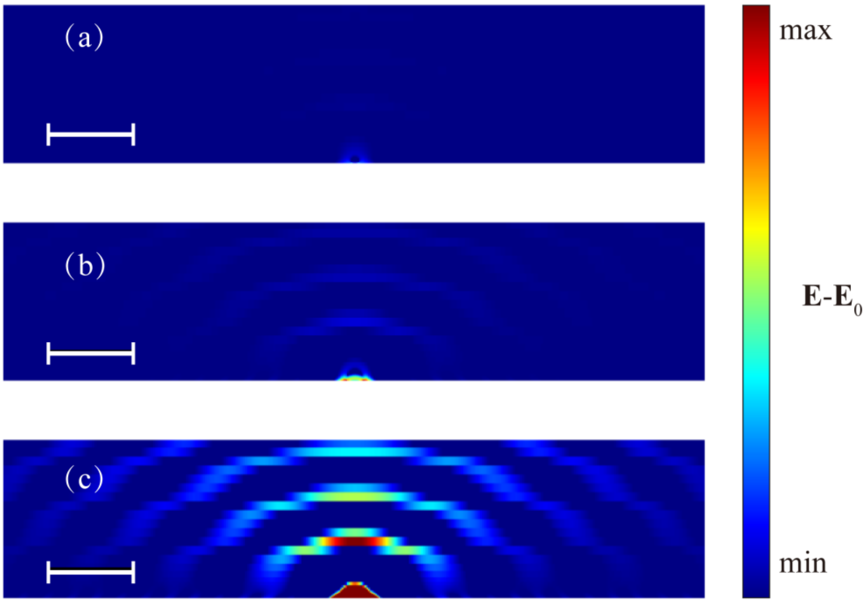

Figure 2 Intensity of quasi-cylindrical waves (y-z plane) excited by a 200 nm thick a-Si film on a c-Si substrate when the oxide particles have radii of **(a)** 50 nm, **(b)** 100 nm and **(c)** 200 nm. The scale bar was 1 μ m.

References

[1] Palik, Edward D., ed. *Handbook of optical constants of solids*. Vol. 3. Academic press, 1998.
